# Epidemiological and clinical characteristics of symptomatic hereditary transthyretin amyloid polyneuropathy: a global case series

**DOI:** 10.1186/s13023-019-1000-1

**Published:** 2019-02-08

**Authors:** Márcia Waddington-Cruz, Hartmut Schmidt, Marc F. Botteman, John A. Carter, Michelle Stewart, Markay Hopps, Shari Fallet, Leslie Amass

**Affiliations:** 1 Federal University of Rio de Janeiro, Rio de Janeiro, Brazil; 2 Muenster University Hospital, Muenster, Germany; 3 Pharmerit International, Bethesda, MD USA; 4 BluePoint, LLC, Chicago, IL USA; 5 Pfizer Inc., Groton, CT USA; 6 Pfizer Inc., New York, NY USA; 7 Pfizer Inc., Collegeville, PA USA; 8 Hospital Universitário Clementino Fraga Filho (HUCFF), Universidade Federal do Rio de Janeiro (UFRJ), Rio de Janeiro, Brazil

**Keywords:** Transthyretin amyloidosis, Peripheral neuropathy, Case series, Rare disease

## Abstract

We describe 542 cases of symptomatic hereditary transthyretin amyloid polyneuropathy (ATTR-PN) identified through a review of the literature published between 2005 and 2016. Approximately 18% of the cases were from countries where ATTR-PN is traditionally considered to be endemic (i.e., Portugal, Japan, and Sweden). East Asia (Japan, China, Taiwan, and South Korea) contributed a sizeable combined proportion (37.0%, *n* = 200) with Japan (*n* = 92) and China (*n* = 71) being the primary contributors. The most common genotypes among the 65 genotypes represented in the sample were Val30Met (47.6%), Ser77Tyr (10%), Ala97Ser (6.5%), and Phe64Leu (4.4%). Cases with genotypes other than the aforementioned four had the lowest ages at onset (mean 49.2 [standard deviation {SD} 21.0; inter-quartile range {IQR}14.7]) and diagnosis (mean 53.4 [SD 21.0; IQR 14.7]). Conversely, Phe64Leu mean age of onset was 67.5 (SD 8.8; IQR 5.2) and mean age of diagnosis was 71.3 (SD 8.8; IQR 5.4). The prevalence of upper and lower limb involvement at the time of diagnosis (67 and 41%) observed across all cases is consistent with the typical presentation of ATTR-PN. Other notable findings at the time of diagnosis included a high rate of impotence among the Ala97Ser cases versus all others (67% vs. 21%) and a high rate of non-motor visual symptoms (i.e., visual opacities and glaucoma) in the Ser77Tyr cases versus all others (93% vs. 16%). Though comparisons were made descriptively and were hindered by inconsistency of reporting across the cases, these findings support the notion that ATTR-PN is a more phenotypically and geographically variable disease than is typically considered.

**Electronic supplementary material:**

The online version of this article (10.1186/s13023-019-1000-1) contains supplementary material, which is available to authorized users.

## Introduction

Transthyretin amyloid polyneuropathy (ATTR-PN) is a rare genetic disease considered to be endemic to Portugal, Sweden, and foci in Japan [1]. Its global prevalence is traditionally and somewhat anecdotally estimated as 5000 to 10,000 [2, 3], but a recently published analysis reported that global prevalence may be as high as 38,000 persons [4]. In ATTR-PN, misfolded amyloid deposits accumulate on the peripheral nerves and within major organs leading to progressive debilitating sensorimotor polyneuropathy and autonomic dysfunction [5]. This may be manifested by motor impairment, muscle weakness and wasting, and multiple organ failure, but the disease is phenotypically heterogeneous [6]. In nearly all cases ATTR-PN will progress and lead to loss of bodily function, diminished quality of life, and death within approximately 10–15 years after onset, often due to cardiac complications [7–11].

Much of what is known about ATTR-PN has been gathered from the study of the most common genotype, Val30Met (i.e., substitution of valine for methionine in position 30 of the transthyretin protein), which in 1984 was the first causative mutation to be identified [12]. The clinical course of ATTR-PN in endemic countries where Val30Met predominates typically consists of symptom onset with sensory-motor symptoms. Some patients may also present with autonomic neuropathy with or without sensory-motor involvement. The age of onset in endemic regions such as Portugal and Brazil generally occurs in the mid-30’s or 40’s while in Sweden onset is much later (age 60–70 years). Similar to Swedish patients, in non-endemic countries, patients with the Val30Met mutation may experience the onset of symptoms at a later age [13]. Thus, in just this one genotype there are clinically important differences in age of onset and how ATTR-PN is expressed. Nearly 100 ATTR genotypes have been identified across approximately 40 countries [4, 13, 14]. This along with the associated phenotypic variability underscores the heterogeneity of this rare disease.

Partly due to this heterogeneity, a knowledge gap exists with regard to recognizing ATTR-PN, particularly in non-endemic countries where the prevalence of non-Val30Met genotypes is greater, which has led to delayed or under-diagnosis and ultimately to suboptimal treatment outcomes [15]. Although likely largely driven by lack of clinician experience and insufficient patient access to specialized treatment centers, the knowledge gap may also be attributable to a lack of consolidated case information in the literature. Published information is often specific to a single geography, institution, or genotype, making it difficult to gain insights into commonalities and differences. We conducted a broad review and synthesis of existing reports of ATTR-PN cases in an effort to develop a more comprehensive view of the clinical presentation of this disease with respect to its sensorimotor characteristics.

## Methods

### Literature review

A previously reported systematic review conducted according to modified Preferred Reporting Items for Reviews and Meta-Analyses (PRISMA) [16] guidelines was used to identify and synthesize ATTR-PN prevalence information globally [4]. The systematic review included structured searches of the peer-reviewed literature published from 2005 to 2016 (inclusive) via the following online reference databases: Embase, PubMed, SCOPUS, and Web of Science. Additionally, the proceedings of the following five conferences were reviewed: (a) First European Congress on Hereditary ATTR Amyloidosis (ATTR 2015); (b) International Society of Amyloidosis (ISA 2010, 2012, and 2014); (c) International Symposium on Familial Amyloidotic Polyneuropathy (ISFAP 2013).

These searches were conducted without regard to language or geography. While conducting the review of prevalence information, individual ATTR-PN clinical cases were identified and retained for further analysis.

### Case eligibility, data extraction, and analysis

**Fig. 1:**
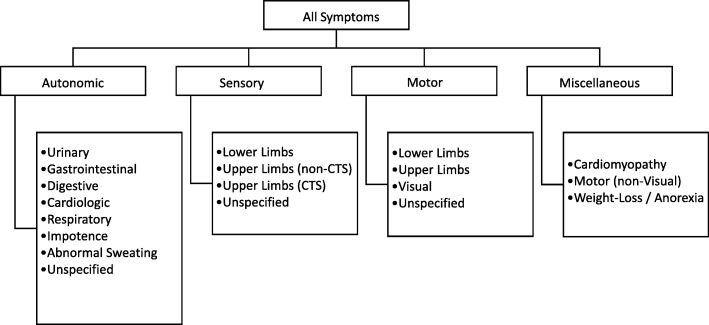
This figure depicts the taxonomy of ATTR-PN symptoms extracted for analysis CTS is carpal tunnel syndrome

For each case identified, data for the following variables were collected and constituted the minimum threshold for retention in the database as a case: (A) confirmation of symptomatic ATTR-PN manifested by explicitly noted polyneuropathy, (B) sex, (C) mutation, and location / country. The following were also extracted where reported: age of (E) symptom onset, (F) diagnosis, (G) death, symptoms at (H) onset, and (I) diagnosis, and (J) parent-of-origin effect (genotypically confirmed). Reported symptoms ascribed in the reports as being of a neuropathic nature were further categorized as autonomic, sensory, motor, and miscellaneous (i.e., cardiomyopathy, motor (non-visual), and weight-loss/anorexia) according to the taxonomy depicted in Fig. 1. Duplicate cases were identified by overlap of variables B-I and subsequently removed, as were cases of de novo disease subsequent to liver transplantation. Descriptive analysis of extracted case data addressed the following:

- Distribution of ATTR-PN genotypes by country of origin
- Age at disease milestones (onset, diagnosis, death) by genotype
- Symptoms reported at initial presentation by genotype

To address anticipated skewness, the mean, standard deviation (SD), and inter-quartile range (IQR) were calculated for disease milestone outcomes using only the values between the first and third quartiles of the extracted data. Time between disease milestones was calculated across only the cases with both values reported.

## Results

There were 653 cases extracted from the literature initially. After applying eligibility criteria and removing duplicative reports, 111 cases from 15 reports were excluded. Seventy exclusions (63%) were due to sex not having been reported, while 28 (25%) of the cases were excluded because they were described as “asymptomatic” and/or no neuropathic symptoms were explicitly described. Seventeen genotypes were represented in the excluded cases, of which 62% were Val30Met followed by 11% Gly83Arg.

The retained sample included 542 cases contributed by 108 individual reports across 32 countries (Table 1). Approximately 18% of the cases were from countries where ATTR-PN is traditionally considered to be endemic (i.e., Portugal, Japan, and Sweden) [17]. Most cases were from Western Europe and the Asia-Pacific region, specifically East Asia. The four most common genotypes among the 65 genotypes represented in the sample were Val30Met (47.6%), Ser77Tyr (10%), Ala97Ser (6.5%), and Phe64Leu (4.4%) (Table 1, Additional file 1: Appendix A). Val30Met was the most prevalent genotype reported in endemic countries, whereas genotypes from non-endemic countries primarily belonged to the “Other” category (i.e., those comprising < 4% of the retained cases). There was insufficient data to assess genotypically-confirmed parent-of-origin effect. Ages at onset of neuropathy, diagnosis, and death were reported for *n* = 394, *n* = 276, and *n* = 139 cases, respectively. Summary statistics for these milestones are listed in Table 2. It was difficult to draw meaningful inter-genotype comparisons from these data due to the heterogeneous nature of the reporting, and because assessment of time from onset or diagnosis to death was biased by right-censoring.

**Table 1:** Global Distribution of Reviewed ATTR-PN Cases, n (%)

Country	Country Total	Ala97Ser	Phe64Leu	Ser77Tyr	Val30Met	Other
France	97 (17.9%)	0 (0%)	0 (0%)	33 (34%)	47 (48.5%)	17 (17.5%)
Japan	92 (17%)	0 (0%)	0 (0%)	0 (0%)	76 (82.6%)	16 (17.4%)
China	71 (13.1%)	2 (2.8%)	0 (0%)	12 (16.9%)	23 (32.4%)	34 (47.9%)
Italy	58 (10.7%)	0 (0%)	24 (41.4%)	0 (0%)	21 (36.2%)	13 (22.4%)
Taiwan	35 (6.5%)	33 (94.3%)	0 (0%)	1 (2.9%)	0 (0%)	1 (2.9%)
Germany	26 (4.8%)	0 (0%)	0 (0%)	0 (0%)	13 (50%)	13 (50%)
Spain	19 (3.5%)	0 (0%)	0 (0%)	0 (0%)	8 (42.1%)	11 (57.9%)
Portugal	18 (3.3%)	0 (0%)	0 (0%)	0 (0%)	16 (88.9%)	2 (11.1%)
Greece	17 (3.1%)	0 (0%)	0 (0%)	0 (0%)	17 (100%)	0 (0%)
Israel	17 (3.1%)	0 (0%)	0 (0%)	8 (47.1%)	4 (23.5%)	5 (29.4%)
Sweden	14 (2.6%)	0 (0%)	0 (0%)	0 (0%)	5 (35.7%)	9 (64.3%)
Ireland	12 (2.2%)	0 (0%)	0 (0%)	0 (0%)	0 (0%)	12 (100%)
Argentina	11 (2%)	0 (0%)	0 (0%)	0 (0%)	8 (72.7%)	3 (27.3%)
Turkey	11 (2%)	0 (0%)	0 (0%)	0 (0%)	4 (36.4%)	7 (63.6%)
Brazil	8 (1.5%)	0 (0%)	0 (0%)	0 (0%)	8 (100%)	0 (0%)
United States	6 (1.1%)	0 (0%)	0 (0%)	0 (0%)	1 (16.7%)	5 (83.3%)
Australia	3 (0.6%)	0 (0%)	0 (0%)	0 (0%)	0 (0%)	3 (100%)
Belgium	3 (0.6%)	0 (0%)	0 (0%)	0 (0%)	3 (100%)	0 (0%)
Finland	3 (0.6%)	0 (0%)	0 (0%)	0 (0%)	0 (0%)	3 (100%)
Poland	3 (0.6%)	0 (0%)	0 (0%)	0 (0%)	0 (0%)	3 (100%)
Romania	3 (0.6%)	0 (0%)	0 (0%)	0 (0%)	0 (0%)	3 (100%)
Korea (South)	2 (0.4%)	0 (0%)	0 (0%)	0 (0%)	0 (0%)	2 (100%)
Russia	2 (0.4%)	0 (0%)	0 (0%)	0 (0%)	0 (0%)	2 (100%)
Slovenia	2 (0.4%)	0 (0%)	0 (0%)	0 (0%)	0 (0%)	2 (100%)
Switzerland	2 (0.4%)	0 (0%)	0 (0%)	0 (0%)	1 (50%)	1 (50%)
Czech Republic	1 (0.2%)	0 (0%)	0 (0%)	0 (0%)	0 (0%)	1 (100%)
Denmark	1 (0.2%)	0 (0%)	0 (0%)	0 (0%)	1 (100%)	0 (0%)
Holland	1 (0.2%)	0 (0%)	0 (0%)	0 (0%)	1 (100%)	0 (0%)
India	1 (0.2%)	0 (0%)	0 (0%)	0 (0%)	0 (0%)	1 (100%)
Malaysia	1 (0.2%)	0 (0%)	0 (0%)	0 (0%)	0 (0%)	1 (100%)
Norway	1 (0.2%)	0 (0%)	0 (0%)	0 (0%)	1 (100%)	0 (0%)
United Kingdom	1 (0.2%)	0 (0%)	0 (0%)	0 (0%)	0 (0%)	1 (100%)
Genotype Total	542 (100%)	35 (6.5%)	24 (4.4%)	54 (10%)	258 (47.6%)	171 (31.5%)

Specific genotypes shown are those with ≥4% representation among the included cases. Genotypes with < 4% representation are listed as “Other”. Refer to Additional file 1: Appendix A for a list of genotypes included in the “Other” category

**Table 2:** Characteristics of 542 ATTR-PN cases

	All	Ala97Ser	Phe64Leu	Ser77Tyr	Val30Met	Other
N	542	35	24	54	258	171
% of Sample	(100%)	(6.5%)	(4.4%)	(10.0%)	(47.6%)	(31.5%)
% from Endemic^a^	17.9%	0.0%	0.0%	0.0%	37.6%	15.8%
% Male	68.6%	85.7%	79.2%	74.1%	69.0%	61.4%
Disease Milestones Mean (SD, IQR) [years]						
Onset	61.5 (11.5; 8.4)	58.5 (8.0; 6.4)	67.5 (8.8; 5.2)	51.6 (12.0; 8.2)	64.0 (12.0; 8.1)	49.2 (21.0; 14.7)
Diagnosis	64.2 (13.6; 9.6)	58 (2.0; 2.2)	71.3 (8.8; 5.4)	57.7 (9.5; 6.4)	68.1 (8.1; 6.7)	53.4 (21.0; 14.7)
Death	66.3 (14.0; 9.9)	–	–	58.5 (5.8; 4.2)	71.0 (9.5; 7.4)	65.7 (15.3; 10.1)
Onset to Diagnosis	2.9 (3.2; 2.1)	8.6 (3.0; 2.1)	3.8 (2.0; 1.8)	2.3 (2.7; 1.5)	3.0 (3.4; 2.4)	2.7 (3.0; 2.2)
Diagnosis to Death	1.9 (2.0; 1.4)	–	–	1.1 (1.1; 0.6)	2.1 (1.9; 1.3)	2.1 (1.8; 1.4)
Onset to Death	5.0 (3.0; 2.4)	–	–	3.9 (2.8; 2.1)	5.9 (4.0; 2.8)	5.4 (2.8; 1.8)

Specific genotypes shown are those with ≥4% representation among the included cases. Genotypes with < 4% representation are listed as “Other”. Refer to Additional file 1: Appendix A for a list of genotypes included in the “Other” category. Refer to Additional file 1: Appendix B for data used to generate disease milestone outcomes. IQR is inter-quartile range. SD is standard deviation

^a^Japan, Portugal, and Sweden were categorized as endemic countries

Referring to Table 2, cases with genotypes in the “other” category had the lowest ages at onset (Mean 49.2 [SD 21.0; IQR 14.7]) and diagnosis (Mean 53.4 [SD 21.0; IQR 14.7]). Conversely, Phe64Leu mean age of onset was 67.5 (SD 8.8; IQR 5.2) and mean age of diagnosis was 71.3 (SD 8.8; IQR 5.4). The mean age of death (uncorrected for censoring and individual case characteristics) for Ser77Tyr was the lowest among all groups (Mean 58.5 [SD 5.8; IQR 4.2]).

Table 3 lists the proportions of cases with given symptoms reported at diagnosis stratified by genotype. Eighty-seven percent of all cases reported sensory neuropathy at diagnosis [18]. Note that all cases retained for this analysis were confirmed by the reporting author(s) to be diagnosed with ATTR-PN and were explicitly described as having experienced sensory neuropathy during the course of their disease that was attributable to ATTR-PN. The methods used to establish these diagnoses were not recorded for the present review. In many reports, the cases received an initial diagnosis after presenting with prolonged gastrointestinal symptoms or abnormal cardiologic findings (e.g., arrhythmia and other cardiac autonomic abnormalities). Among the cases reporting sensory neuropathy at the time of diagnosis, more had lower limb versus upper limb involvement (67% vs. 41%), which is consistent with the characterization of ATTR-PN sensory neuropathy originating in the feet and later spreading to the upper limbs as the disease progresses [19]. Other notable findings at the time of diagnosis included a relatively high rate of impotence among the Ala97Ser cases versus all others (67% vs. 21%) and a high rate of non-motor visual symptoms (i.e., visual opacities and glaucoma) in the Ser77Tyr cases versus all others (93% vs. 16%).

**Table 3:** Clinical Characteristics at Presentation

Feature	All	Ala97Ser	Phe64Leu	Ser77Tyr	Val30Met	Other
Any Reported	374 (69%)	35 (9%)	22 (6%)	48 (13%)	141 (38%)	128 (34%)
Autonomic	199 (53%)	27 (77%)	18 (82%)	5 (10%)	74 (52%)	75 (59%)
Unspecified	47	0	7	1	22	17
Urinary	36	7	3	0	16	10
Gastrointestinal	114	22	6	3	39	44
Cardio	96	15	7	1	34	39
Respiratory	3	0	0	0	0	3
Impotence	51	18	5	1	13	14
Sweat	20	7	3	1	4	5
Sensory	326 (87%)	25 (71%)	22 (100%)	45 (94%)	127 (90%)	107 (84%)
Unspecified	76	21	5	0	25	25
Lower Limbs	219	2	15	37	100	65
Upper Limbs	133	2	10	35	48	38
Carpal Tunnel	42	4	4	0	7	27
Motor	215 (57%)	10 (29%)	15 (68%)	32 (67%)	79 (56%)	79 (62%)
Unspecified	67	9	4	0	33	21
Lower Limbs	138	0	11	28	46	53
Upper Limbs	83	1	10	10	33	29
Other	1	0	0	0	0	1
Miscellaneous	155 (41%)	4 (11%)	17 (77%)	14 (29%)	39 (28%)	81 (63%)
Cardiomyopathy	93	4	15	2	28	44
Visual (Non-Motor)	49	0	2	13	9	25
Weight Loss	35	0	2	2	9	22

Specific genotypes shown are those with ≥4% representation among the included cases. Genotypes with < 4% representation are listed as “Other”. Refer to Additional file 1: Appendix A for a list of genotypes included in the “Other” category

## Discussion

A search of bibliographic databases and the proceedings of amyloidosis-focused clinical conferences yielded 542 unique ATTR-PN cases. Four genotypes (Val30Met, Ser77Tyr, Ala97Ser, and Phe64Leu) comprised 70% of the total case sample, while 65 genotypes were identified overall. France was the largest single locus of cases (17.9%, *n* = 97) among 32 countries represented; however, East Asia (Japan, China, Taiwan, and South Korea) contributed a sizeable combined proportion (37.0%, *n* = 200) with Japan (*n* = 92) and China (*n* = 71) being the primary contributors. The remaining 245 cases originated mostly from Western Europe: particularly Italy (*n* = 58) and Germany (*n* = 26).

There were notable findings with regard to the timing of key disease milestones (i.e., onset, diagnosis, and death). For example, the mean age of symptom onset across ATTR-PN cases included here was 61.5 (±11.5) years, whereas traditionally, disease onset has been reported to occur by age 50 [15, 20, 21]. Seemingly dissonant findings are explained by examining the characteristics of cases in previous reports versus the present one. Published estimates for the timing of disease milestones are heavily influenced by patients with Portuguese-type Val30Met disease in endemic countries, which is the most prevalent form and for which onset is typically at 30–40 years old [15], and generally onset is earlier in endemic versus non-endemic countries (except in Sweden where it is typically late-onset). Furthermore, previous case series have reported that age of onset is later for non-Val30Met ATTR-PN [21, 22]. Thus, the later onset reported here appears consistent with the aforementioned trends given that cases were predominantly from non-endemic countries and non-Val30Met (Table 2).

The relatively higher onset age reported here might also reflect changing trends in disease characteristics at presentation that have been influenced by better disease awareness and population factors such as decreasing fertility rates. In their recently published epidemiological assessment of ATTR-PN in Portugal, Ines et al. (2018) implicated these same factors as likely reasons for a higher incident age [23]. The authors noted that the ratio of late-onset to early-onset incident cases nearly doubled from 1:4 (22.4%) to 2:4 (44.4%) between 2010 and 2016. They ascribed this to the upward influence on late-onset diagnoses generated by better late-onset disease recognition, and the downward influence on early-onset cases generated by a 50% decline in the national fertility rate over the past 40 years. This trend will likely accelerate as genetic counseling and medical-assisted reproductive methods instituted over the past three decades begin to have a more demonstrable effect in reducing carrier prevalence [23].

After recognizing that the cases included here were atypical in the sense that they were generally later-onset and non-Val30Met, it was also important to examine how this might be related to the observed time between onset and death. The reported mean time from symptom onset to death in persons with ATTR-PN is 10–15 years [14, 24]. In the present review this value was 5 years. This descriptive assessment did not adjust for censoring or case characteristics, and the potential biasing effects cannot be overlooked. However, this discrepancy between average values in the literature and our findings may be somewhat attributable to geographic origin and genotype. Our sample was largely atypical and sporadic. As has been noted previously, sporadic cases may not receive adequate treatment as early as more typical cases (i.e., those presenting in endemic areas with Val30Met disease). Variable clinical features combined with limited physician awareness and insufficient diagnostic capabilities in non-endemic areas may have resulted in delayed diagnosis and treatment, which would expedite disease progression and death [25]. With regard to our analysis of symptoms at onset and diagnosis, we observed a typical progression [26] overall in that sensory disturbances generally originated in the distal lower extremities and spread proximally. The contention that our sample represents a more sporadic and later-onset population in which diagnosis was delayed is supported by observed relatively high rates of motor and autonomic dysfunction, both hallmarks of progressed disease [26, 27]. This coupled with the observed average age of symptom onset and signs of progressed disease at diagnosis provide further support for the conclusion that these cases were generally late-onset and received delayed diagnoses. It is also notable that many cases were initially diagnosed incorrectly with chronic inflammatory demyelinating polyneuropathy, which is consistent with previously reported cases described as sporadic and late-onset [20].

While this case series provides useful information regarding the genotypic, phenotypic, and geographic variability of ATTR-PN, our descriptive analysis was limited by inconsistency among the individual case reports. For example, in 13% of cases there was no description of sensory neuropathy despite all cases having reportedly been diagnosed with symptomatic ATTR-PN for which sensory neuropathy is the most common initial symptom. Furthermore, the sample size was not sufficient to draw statistically robust comparisons among genotypes for the timing of disease milestones and the composition of symptoms at onset/diagnosis. It is also possible that selective reporting of novel mutations and sporadic cases may have biased the results, and noted differences in symptoms might reflect differences in data collection rather than disease presentation. Lastly, this review was limited to the sensorimotor characteristics of ATTR-PN, but this is not a complete clinical picture of the disease, particularly in countries like the United States and United Kingdom where cardiac involvement – namely heart failure with preserved ejection fraction – is the predominant presenting characteristic for the hereditary form of the disease.

Despite some limitations, these case reports are an important resource for this rare, progressive, and generally fatal disease. Non-endemic regions for example have few patients, but disproportionately many of them are sporadic cases for which a positive family history of ATTR-PN – typically among the most apparent risk factors [27] – is either lacking or not evaluable to facilitate timely diagnosis. This point cannot be overstated because without adequate information, the pattern of sensory-motor and autonomic neuropathy in patients with early ATTR-PN who would benefit most from treatment may be indistinguishable from more common diagnoses [27]. This report is also relevant in endemic areas because it emphasizes that their true ATTR-PN populations likely extend beyond historically predominant genotypes and phenotypes. Overall, knowledge of ATTR-PN appears largely derived from endemic areas and persons with early-onset Val30Met disease because the disease is exceedingly rare otherwise. It is hoped that comprehensive case series such as this will help broaden the understanding of ATTR-PN - providing insight into non-endemic areas and less common genotypes - so that afflicted persons can receive prompt, accurate diagnosis and begin treatment when it will be most effective.

## Additional file

Additional file 1: Appendix A. Genotypes Included in the “Other” Category. Appendix B. Summary Statistics for Disease Milestone Outcomes without Outliers. (DOCX 20 kb)

## Abbreviations

ATTR-PN: Transthyretin amyloid polyneuropathy
IQR: Inter-quartile range
PRISMA: Preferred Reporting Items for Systematic Reviews and Meta-Analyses
SD: Standard deviation
