# Supplementary material for: Epidemiological and clinical characteristics of symptomatic hereditary transthyretin amyloid polyneuropathy: a global case series
Source: Orphanet J Rare Dis. 2019 Feb 8;14:34. doi: 10.1186/s13023-019-1000-1 (PMC6368811; doi:10.1186/s13023-019-1000-1)
Supplement: Supplementary file 1 — Appendix A. Genotypes Included in the “Other” Category. Appendix B. Summary Statistics for Disease Milestone Outcomes without Outliers. (DOCX 20 kb) [file 13023_2019_1000_MOESM1_ESM.docx]

# APPENDIX A. Genotypes Included in the “Other” Category

Ala117Ser Ala120Ser Ala20Ser Ala36Pro Ala45Asp Ala45Ser Arg34Gly Arg34Thr Asp38Ala Asp58Val Glu42Gly Glu54Gln Glu54Lys Glu74Gly Glu81Lys Glu89Gln Glu89Gly Glu89Lys Gly47Arg Gly47Val Gly57Arg Gly67Glu Gly83Arg His110Asp His88Arg Ile107Met Ile107Phe Ile107Val Ile68Leu Leu55Gln Leu55Pro Leu58Arg Leu58His Lle107Val Lys35Asn Lys35Thr Lys90Glu Phe33Leu Phe33Val Phe64Val Ser23Asn Ser50Arg Ser70Arg Thr49Ala Thr49Ser Thr59Arg Thr59Lys Thr60Ala Thr60Ala Tyr114Cys Tyr134His Val122Ala Val122Ile Val30Ala Val30Leu Val32Ala Val50Ala Val93Met

# APPENDIX B. Summary Statistics for Disease Milestone Outcomes without Outliers

| **Age at Onset** | **All** | **Ala97Ser** | **Phe64Leu** | **Ser77Tyr** | **Val30Met** | **Other** |
| --- | --- | --- | --- | --- | --- | --- |
| 3rd Quartile | 66.3 | 63 | 71 | 59 | 70 | 60 |
| 1st Quartile | 54.8 | 55 | 62.3 | 47 | 58 | 39 |
| Inter-Quartile Range | 11.5 | 8 | 8.8 | 12 | 12 | 21 |
| Average | 61.5 | 58.5 | 67.5 | 51.6 | 64 | 49.2 |
| Standard Deviation | 8.4 | 6.4 | 5.2 | 8.2 | 8.1 | 14.7 |
|  |  |  |  |  |  |  |
| **Age at Diagnosis** | **All** | **Ala97Ser** | **Phe64Leu** | **Ser77Tyr** | **Val30Met** | **Other** |
| 3rd Quartile | 71 | 60 | 75 | 62.5 | 72.1 | 64.5 |
| 1st Quartile | 57.4 | 58 | 66.3 | 53 | 64 | 43.5 |
| Inter-Quartile Range | 13.6 | 2 | 8.8 | 9.5 | 8.1 | 21 |
| Average | 64.2 | 58 | 71.3 | 57.7 | 68.1 | 53.4 |
| Standard Deviation | 9.6 | 2.2 | 5.4 | 6.4 | 6.7 | 14.7 |
|  |  |  |  |  |  |  |
| **Age at Death** | **All** | **Ala97Ser** | **Phe64Leu** | **Ser77Tyr** | **Val30Met** | **Other** |
| 3rd Quartile | 73 | -- | -- | 61 | 76 | 72.8 |
| 1st Quartile | 59 | -- | -- | 55.3 | 66.5 | 57.5 |
| Inter-Quartile Range | 14 | -- | -- | 5.8 | 9.5 | 15.3 |
| Average | 66.3 | -- | -- | 58.5 | 71 | 65.7 |
| Standard Deviation | 9.9 | -- | -- | 4.2 | 7.4 | 10.1 |
|  |  |  |  |  |  |  |
| **Onset to Diagnosis** | **All** | **Ala97Ser** | **Phe64Leu** | **Ser77Tyr** | **Val30Met** | **Other** |
| 3rd Quartile | 4.4 | 10 | 5 | 3.7 | 4.5 | 4 |
| 1st Quartile | 1.2 | 7 | 3 | 1 | 1.1 | 1 |
| Inter-Quartile Range | 3.2 | 3 | 2 | 2.7 | 3.4 | 3 |
| Average | 2.9 | 8.6 | 3.8 | 2.3 | 3 | 2.7 |
| Standard Deviation | 2.1 | 2.1 | 1.8 | 1.5 | 2.4 | 2.2 |
|  |  |  |  |  |  |  |
| **Diagnosis to Death*** | **All** | **Ala97Ser** | **Phe64Leu** | **Ser77Tyr** | **Val30Met** | **Other** |
| 3rd Quartile | 3 | -- | -- | 1.7 | 3.1 | 3 |
| 1st Quartile | 1 | -- | -- | 0.7 | 1.2 | 1.2 |
| Inter-Quartile Range | 2 | -- | -- | 1.1 | 1.9 | 1.8 |
| Average | 1.9 | -- | -- | 1.1 | 2.1 | 2.1 |
| Standard Deviation | 1.4 | -- | -- | 0.6 | 1.3 | 1.4 |
|  |  |  |  |  |  |  |
| **Onset to Death*** | **All** | **Ala97Ser** | **Phe64Leu** | **Ser77Tyr** | **Val30Met** | **Other** |
| 3rd Quartile | 7 | -- | -- | 5.8 | 8 | 7.8 |
| 1st Quartile | 4 | -- | -- | 3 | 4 | 5 |
| Inter-Quartile Range | 3 | -- | -- | 2.8 | 4 | 2.8 |
| Average | 5 | -- | -- | 3.9 | 5.9 | 5.4 |

*These outcomes are likely heavily biased due to not being able to control for censoring effects or case characteristics. They should be interpreted with caution. Note: The double dash indicates where values could not be calculated due to insufficient data.
